# A Study on Increasing Sensitivity of Rectangular Microcantilevers Used in Biosensors

**DOI:** 10.3390/s8117530

**Published:** 2008-11-25

**Authors:** Mohd. Zahid Ansari, Chongdu Cho

**Affiliations:** Department of Mechanical Engineering, Inha University, 253 Yonghyun-dong, Nam-Ku, Incheon, 402-751 Republic of Korea; E-Mail: ansari.zahid@hotmail.com

**Keywords:** Surface stress, Biosensor, Microcantilever, Stoney equation, Sader equation

## Abstract

This study proposes a new microcantilever design with a rectangular hole at the fixed end of the cantilever that is more sensitive than conventional ones. A commercial finite element analysis software ANSYS is used to analyze it. The Stoney equation is first used to calculate the surface stress induced moment, and then applied to the microcantilever free end to produce deflection. The stress analysis of the proposed and conventional designs is performed, followed by dynamic analysis of the proposed design. We found that the Sader equation is more accurate than Stoney in predicting cantilever deflections, and that for increasing the sensitivity of a microcantilever biosensor increasing the cantilever thickness is more practical.

## 1. Introduction

Biosensors are electronic devices that convert biomolecular interactions into a measurable signal. The purpose of a biosensor is to detect and analyze the unknown biological elements present in a medium. Biosensors have two main elements, a bioreceptor and a transducer. Bioreceptors are target-specific and known biomolecules that combine with the target analyte molecules, and generate a unique signal during the reaction. For sensing purpose one surface of the biosensor is functionalized by depositing a sensing layer of known bioreceptor molecules onto it. This biosensitive layer either contains the bioreceptors or the bioreceptors are covalently bonded to it. The most common types of bioreceptors used in biosensing are based on proteins, antibody/antigen or nucleic acid interactions. The transducer element of the biosensor converts the biomolecular reactions between the target and bioreceptor molecules into a measurable signal. The signals can be measured using appropriate detection techniques like electrochemical, optical or mechanical. In biosensing applications sample preparation and molecular labelling of the target analyte is a basic requirement. Labelling aids in easy detection and monitoring of the biomolecules and bioreactions progress. Radioactive and fluorescent dye based labelling agents are commonly used in biosensors. Labelling is however an expensive and time consuming process. Therefore, label-free detection technique is critical in developing rapid, economic and user-friendly biosensors and bioanalytical kits.

The ability of label-free detection, scalability to allow massive parallelization, and sensitivity of the detection range applicable to *in vivo* problems are the important requirements for a future generation of biosensors [1]. Surface plasmon resonance (SPR) [2], quartz crystal micro-balances (QCM) [3], and cantilever array biosensors [4, 5] are three such attractive label-free detections techniques. Both SPR and QCM utilize the mass-change induced frequency variations to assay the target analyte. SPR is an optical detection technique which measures the change in the refractive index of the biosensing surface upon the biomolecular interactions. When the target molecules attach onto the functionalized surface the resonance frequency of the surface plasmons is changed, affecting the refractive index of the surface. QCM is a mechanical detection technique which measures the mass change by measuring the change in frequency of a quartz crystal. One surface of the crystal is functionalized with bioreceptor molecules. When target molecules attach onto the mechanical vibration frequency of the crystal is changed.

Although generally used in topological investigations of surfaces such as in the atomic force microscopy (AFM), arrays of microcantilevers are attracting much interest as biosensors in label-free, rapid, and realtime assaying of biomolecules. Microcantilevers are being used in a variety of sensing and diagnostic applications. Sander *et al.* [6] used microcantilevers in measurements of surface stress, surface reconstruction, film stress and magnetoelastic stress of monolayers. Nordstrom *et al.* [7] reported detailed analysis of fabrication and characterization SU-8 microcantilevers for biological and chemical sensing, fabrication. They also developed some novel deflection readout techniques. McKendry *et al.* [5] used an eight cantilevers array to detect unlabeled DNA hybridizations at nanomolar concentrations within minutes. Arntz *et al.* [1] used a similar array for realtime detection of two cardiac biomarkers proteins: myoglobin and kinase, whose level in the blood indicate the presence of acute myocardial infarction, a type of heart disease. Zhang *et al.* [8] successfully employed the microcantilever biosensor in the rapid and labelfree detection of biomarker transcripts in human RNA in picomolar concentration range. Suri *et al.* [9] employed microcantilevers in detecting atrazine, a dangerous pesticide found in agricultural fields, with parts per trillion (ppt) accuracy. Knowles *et al.* [10] used a microcantilever in assaying amyloid growth and protein aggregations. Recently, Mortens *et al.* [11] used an array of eight cantilevers in label-free detection of DNA hybridization based on hydration induced tension in nucleic acid films.

**Figure 1.**
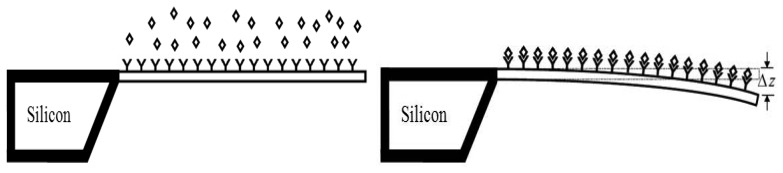
Working principle of a microcantilever biosensor. Functionalization of the biosensor by depositing bioreceptors (left). Surface stress induces deflection (right). Symbols **◊** and **Y** represent target analyte and bioreceptor molecules.

Cantilever array biosensors use optical detection technique to measure the surface-stress induced deflections in a microcantilever. When the target molecules attach to their functionalized surface, the surface stress distribution on the surface is changed causing deflections in the cantilever (Figure 1). During adsorption of target molecules onto the functionalized cantilever surface, biochemical reactions occur which reduces the free energy of the cantilever surface. The reduction in free energy of one side of cantilever is balanced by increase in strain energy of the other side, producing deflection in the cantilever [4, 12]. The deflections may be upward or downward depending on the type of molecules involved and are linearly proportional to the target analyte solution concentration [12]. It means that higher deflections manifest higher sensitivity in the cantilever biosensor. Since the induced surface stress strongly depends on the molecular species and its concentration, by measuring the cantilever deflection the attaching species as well as its concentration can be determined.

With the ability of label-free detection and scalability to allow massive parallelization already realized by microcantilever biosensors, the next challenge in cantilever biosensor development lies is achieving the sensitivity in detection range applicable to *in vivo* analysis. The sensitivity of a cantilever biosensor strongly depends on it ability to convert biochemical interaction into micromechanical motion of the cantilever. The deflections of a cantilever biosensor are usually of the order of few tens to few hundreds of a nanometre. Such extremely low deflections necessitate use of advanced instruments for accurately measuring the deflections. As a consequence, most of the applications of cantilever biosensors are done in laboratories equipped with sophisticated deflection detection and readout techniques. The authors believe that if the deflections of a cantilever biosensor be increased, its advantages will be two fold. First, if the deflections are high a less sensitive readout technique can be used to accurately measure the deflection, which will help in reducing the cost of a cantilever-based biosensor kit. Second, it will help us in detecting analytes in *in vivo* solution concentrations range. The concentrations of some clinically important analytes vary between 10^-4^ to 10^-15^ mol/L. The detection of analytes in such large dynamic range requires an extremely sensitive cantilever. This study proposes and analyses a new high sensitive cantilever design that can assay analytes in extremely low concentrations. A commercial finite element analysis software ANSYS is used to analyze and compare the conventional and the proposed microcantilever designs.

## 2. Mathematical Theory of Cantilever Motion

**Figure 2.**
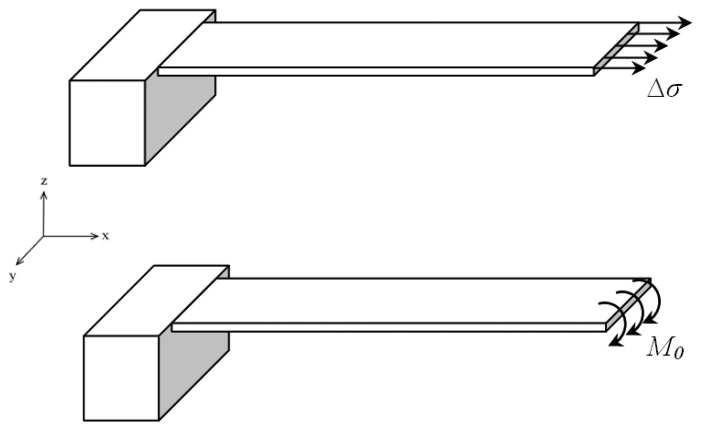
Modelling the surface stress induced curvature in a microcantilever by equating to a concentrated moment induced curvature. The concentrated moment and the surface stress are related as *M_0_* = Δ*σt*/2, where *t* is the thickness of the microcantilever.

The Stoney equation [13] is a fundamental expression relating the residual surface stress (Δ*σ*) per unit length in a film to the curvature (*κ*) of a substrate the film is deposited onto. The curvature does not depend on the material or the geometric properties of the film. This equation is commonly used in determining the residual surface stresses in thin films. In its original form, the equation was given as:

(1) $$\kappa =\frac{6\Delta \sigma }{Et^{2}}$$

where *E* and *t* are the elastic modulus and the thickness of the substrate. Since the cantilever plate is long and wide, in general practice *E* is replaced by the biaxial modulus *E*/(1*- ν*) to accommodate the Poisson ratio (*ν*) coupling. Surface stresses in solids are assumed analogous to the surface tension in liquids. The unit of surface stress measurement is different from that of bulk stress. For the bulk stress it is N/m^2^, whereas for surface stress it is N/m. For modelling purposes the surface stress induced deflection in a substrate is often compared to a concentrated moment induced deflection in a thin plate. Figure 2 shows the schematic for a cantilever plate subjected to a concentrated moment on its free end while the other end is fully constrained.

Applying the Stoney equation assumption that the surface stress bends the plate with uniform curvature, into the concentrated moment induced plate bending the following curvature relation for plate bending can be given as:

(2) $$\kappa =\frac{M_{0}}{EI}$$

where *M_0_* is the applied concentrated moment, *E* is the elastic modulus of the plate, *I* is the moment of inertia of the beam. For a beam of rectangular cross-section the moment of inertia is given as *I* = *bt*^3^/12, where *b* and *t* are the width and the thickness of the beam, respectively. Comparing the curvature relations (1) and (2) the following relation between the surface stress and the moment per unit length can be established:

(3) $$M_{0}=\frac{\Delta \sigma t}{2}$$

This relation shows that the moment is directly proportional to the induced surface stress and the geometric properties of the plate. Moreover, it does not depend on the material properties of the plate. The governing differential equation for an isotropic, thin plates expressing the bending and twisting moments in terms of the curvature and the deflection is given as [14]:

(4a) $$\frac{\partial^{2}z}{\partial x^{2}}+v\frac{\partial^{2}z}{\partial y^{2}}=\frac{M_{x}}{D}$$

(4b) $$\frac{\partial^{2}z}{\partial y^{2}}+v\frac{\partial^{2}z}{\partial x^{2}}=\frac{M_{x}}{D}$$

(4c) $$\frac{\partial^{2}z}{\partial x\partial y}=\frac{M_{xy}}{(1-v)D}$$

where *D* = *E t*^3^/12(1*- ν*^2^) is the flexural rigidity of the plate. In these equations, the moments are expressed in moment per unit length. Assuming *M_x_* = *M_y_* = *M_0_* and neglecting the shear component *M_xy_*, the above equation can be solved to give:

(5) $$z=\frac{\Delta \sigma t(x^{2}+y^{2})}{4D(1+v)}$$

If the cantilever is clamped in such a way that its y-direction motion is restricted, the above can be further simplified as:

(6) $$z=\frac{3(1-v)\Delta \sigma }{E}{\left(\frac{l}{t}\right)}^{2}$$

which is the well known form of Stoney equation commonly used in predicting the residual surface stresses in thin films by measuring the induced deflection. A major assumption made in deriving Equation (6) other than the assumption of uniform curvature is that it is derived for an unrestrained thin plate where all the edges are free to move. In practical applications however at least one edge of the plate must be constrained to get the desired action. Constraining will however violate the assumption of free edges inherent in Equation (6). To accommodate the clamped edge boundary condition Sader [15] proposed a modified Stoney equation:

(7) $$z=\frac{3K(1-v)\Delta \sigma }{E}{\left(\frac{l}{t}\right)}^{2}$$

where *K* is a constant dependent on the material and geometric properties of the cantilever. For a cantilever *o*f *l/b***>** 5 and **v <** 0.25, the value of *K* lies between 1 and 1.05.

**Figure 3.**
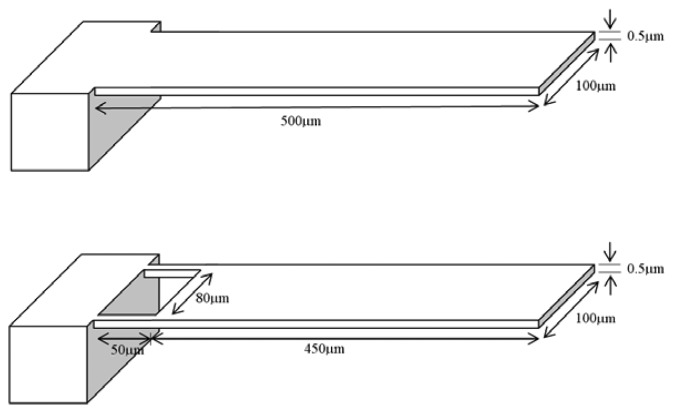
Geometric models of the conventional (upper) and the proposed (lower) microcantilever designs. The material properties and the thickness of them are identical.

Figure 3 shows the conventional and the proposed microcantilever designs. The total lengths and the thicknesses of the two designs are same. The proposed design has two narrow strips towards the fixed end. Each strip is 50 μm long and 10 μm wide. A finite element analysis software ANSYS is used to analyze the deflection and stress behaviour of both the microcantilever designs. For analyzing the deflection behaviour of the cantilever, a moment per unit length of 1.25 × 10^-8^ Nm/m, calculated from Equation 3, is applied at their free ends.

## 3. Results

This study used the microcantilever properties and the experimental data reported in Arntz *et al.* [1] as a reference model. Using an array of eight conventional microcantilevers Arntz *et al.* [1] reported that a maximum surface stress of 0.05 N/m is generated upon injection of 50 μg mL^-1^(∼2.5 μM) myoglobin protein onto the functionalized surface of the silicon microcantilever, which generated a maximum deflection of 0.89 μm at the free end. The cantilever size was 500×100×0.5 μm, and the elastic modulus and Poisson ratio was 130 GPA and 0.28, respectively. The deflections predicted by Stoney equation and the Sader formula can be calculated straightforwardly. In Sader formula (Equation 7) the constant *K* can be calculated from [15]. We found *K* =1.04 for the above microcantilever.

Table 1 compares the analysis results with the experimental result for the surface stress induced maximum deflection in a conventional microcantilever biosensor. The simulation result using moment relation (Equation (3)) shows good accord with the experimental result in [1]. Comparing the experimental results with those predicted by Stoney and Sader relations we clearly observe that the value predicted by Sader is closer to the experimental. It implies that Sader relation is more accurate than the Stoney in predicting the microcantilever deflection. Furthermore, the good accord between Equation (3) and experimental result validates the assumption that the surface stress induced curvature can be equated to the concentrated moment induced curvature in a microcantilever. The simulations result for deflection will be used next in comparing the sensitivity of the proposed design.

**Figure 4.**
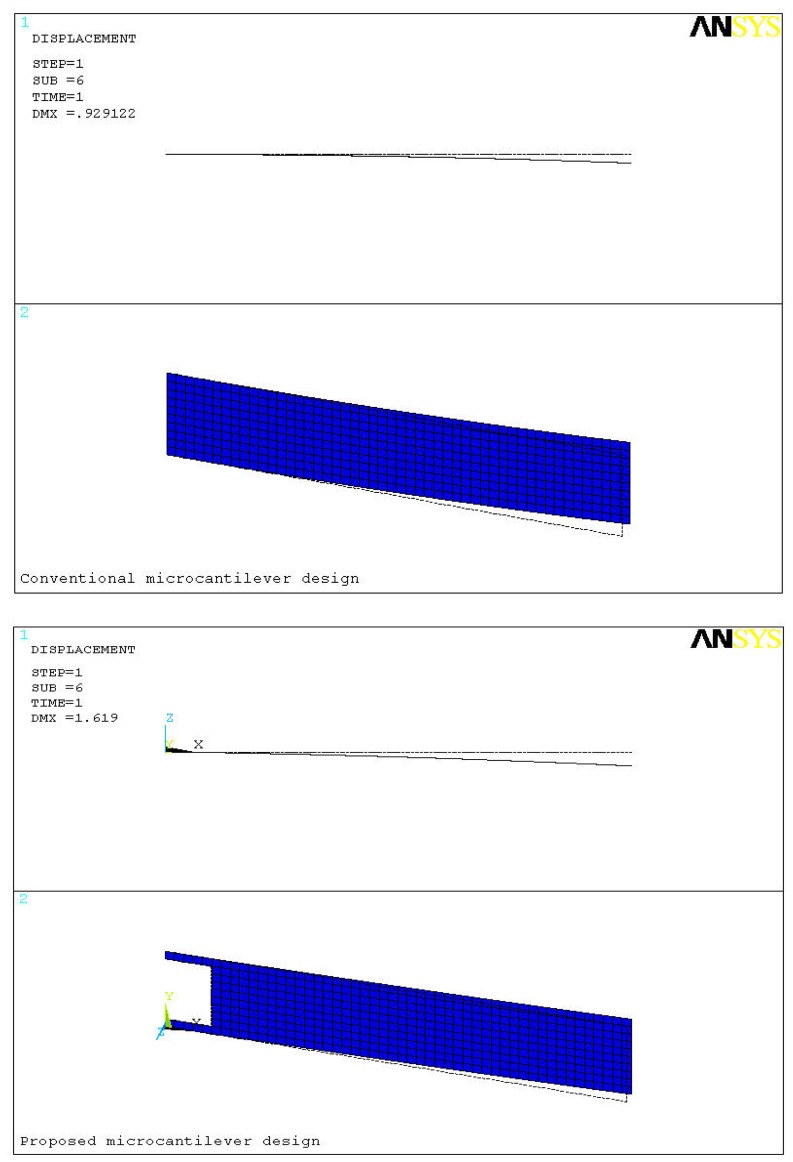
Maximum deflections produced in the conventional (upper) and the proposed (lower) designs. The deformed finite element model is shown in blue. For illustration the deflections are displayed scaled up 10-fold.

Figure 4 shows the comparison between conventional and proposed microcantilever designs. The deformed and undeformed shape of the microcantilever is also marked in the figure. In the analysis of the conventional and proposed cantilevers, respectively 500 and 460 4-node SHELL181 elements were used. The dotted lines show the undeformed shape. The size units in the figure are in micrometers. For a concentrated moment of 1.25 × 10^-8^ Nm/m applied at the free end of the microcantilever deflections of 0.93 μm and 1.62 μm are produced in the conventional and the proposed designs, respectively. In other words, the deflection in the proposed design is about 75% more than that in the conventional design. Since the sensitivity of a microcantilever biosensor strongly depends on its efficient transformation of biomolecular interactions into the deflection, higher deflection produced in the cantilever indicate higher sensitivity of the biosensor. Based on this comparison we can say that the new design is about 75% more sensitive than the old one. In the figure since the deflection is negligible compared to the overall dimensions of the cantilever, deflection is indistinguishable in the plot. Therefore, for clear representation the deflections shown in Figure 4 are scaled up 10-fold. The deflection magnitudes are however unchanged.

**Figure 5.**
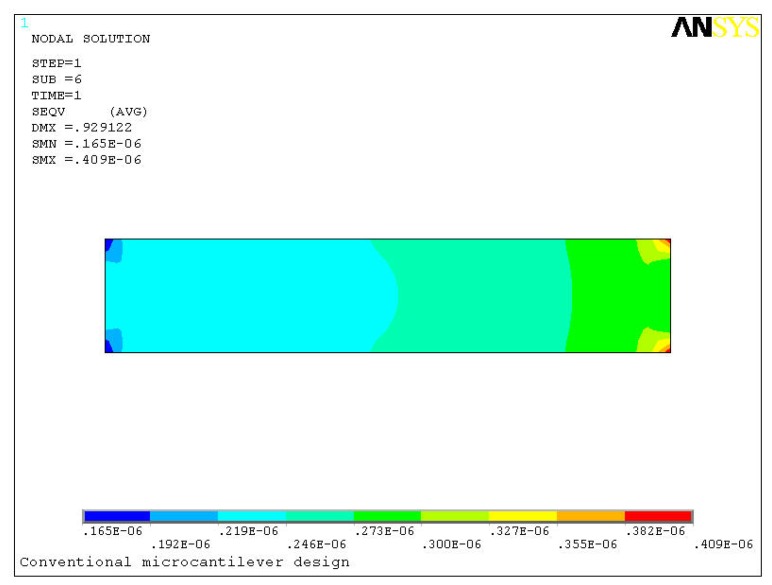

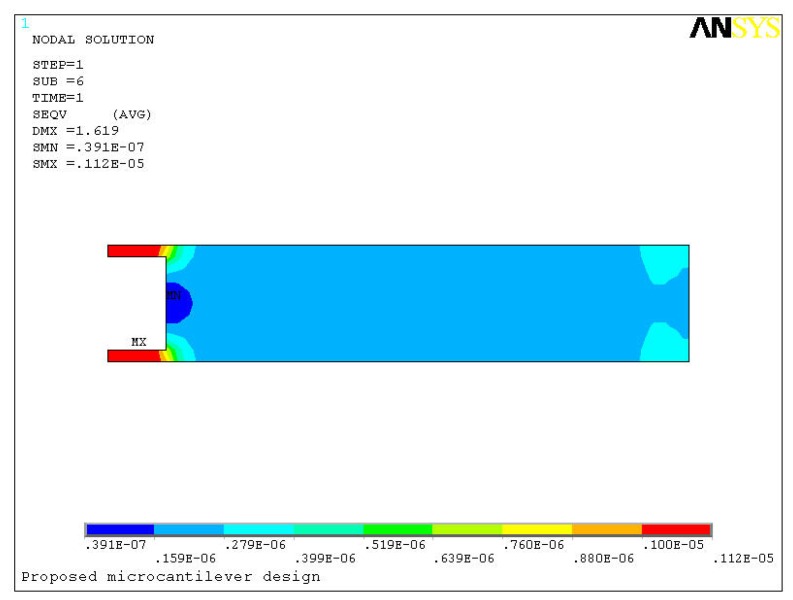
Von Mises stress distribution in the conventional (upper) and the proposed (lower) designs.

Figure 5 shows a comparison between the stresses induced in the two designs due to the bending. For deflections of 0.93 μm and 1.62 μm in the two designs, the maximum induced stresses are 0.41 MPa and 1.12 MPa, respectively. In other words, the new design produced about three times higher stress than the conventional design. In the proposed design the maximum stress is generated in the narrow strips towards the fixed end. The higher stress induced in the new design is understandable because the high deflection in the new design will definitely induce higher stress near the fixed end. In reality however this apparent high stress is trivial because microcantilevers are generally made of silicon, which is an excellent structural material. The elastic modulus and ultimate strength of silicon is around 130 GPa and 7 GPa, respectively [16].

The sharp corners in the proposed microcantilever however can raise the stress concentration factors by many folds. Although in theory the ultimate strength of silicon is very high, but in practice it is of the order of 300 MPa, because of the sharp corners introduced by anisotropic etching [17]. Therefore, an ultimate strength of 300 MPa is more practical for the proposed microcantilever design. In our analysis the corners increased the maximum stress about three times. Since the maximum induced stress of 1.12 MPa is still much lower compared to the ultimate strength of 300 MPa, we can conclude the new design is safe and will not fail under normal conditions.

## 4. Discussion

From the Stoney equation it is clear that for a given surface stress the induced deflection and hence the sensitivity of a cantilever sensor can be increased by either changing the cantilever material or the cantilever geometry. In other words, the equation suggests that by using a cantilever of low elastic modulus material, the deflection can be increased. Since the amount of deflection produced in a microcantilever is linearly proportional to the target analyte solution concentration, higher deflection is an indicator of higher sensitivity. Zhang and Xu [18] fabricated cantilevers using polyethylene terephthalate (PET) films. Others experimented with a polymeric cantilever material SU-8, an epoxy based negative photoresist [19-21]. The polymer cantilevers have certain advantage over silicon cantilevers in being soft, cheap and easy to fabricate. The main advantage in using polymer cantilevers lies in its low Young's modulus, generally about 5 GPa. In contrast the silicon cantilevers has much higher modulus of around 130 GPa. Nevertheless as biosensors the polymer cantilevers have a major limitation: bimetallic thermal effects.

In biological sensor applications the microcantilevers are usually coated with thin film of gold. The gold film helps formation of monolayer of bioreceptors onto it during functionalization. The film also acts as a reflecting medium during optical readout. Since the thermal conductivity of the gold film is much higher than the silicon or polymer cantilever substrate, thermal stresses are generated which produce bimetallic effects. Investigating the bimetallic effects by depositing a 20 nm thick gold layer on a 1 μm thick silicon substrate cantilever, Ramos *et al.* [22] reported that for a temperature change in range of -30 ∼ 25 K the induced thermal deflections decreased linearly with temperature at a rate of 43nm/K. Similarly, Ransley *et al.* [20] reported that gold-coated SU-8 microcantilevers have poor structural integrity and their large thermal expansivity necessitates fine control of the temperature. Thus, both silicon and polymer cantilevers necessitate a fine control of the ambient temperature. The thermal sensitivity of silicon cantilevers is lower than polymer cantilevers because though there is a big difference between the thermal conductivities of gold and silicon, the elastic modulus of silicon is about two times of gold. Therefore, though measurable the thermal deflections in the silicon cantilevers can be neglected. Nevertheless these deflections produce noise in the readout results and hence strongly affect the cantilever sensing accuracy. To avoid the thermal induced deflections in the measurements, differential readout techniques are commonly used. In this technique, one cantilever is made passive by depositing buffer materials onto it and hence it does not participate in analyte-receptor reactions. This cantilever is then set as a reference, and its magnitude of its deflection is deducted from the deflections of other cantilevers. Thus, we have deflections induced due to the surface-stress change only.

We can also reduce bimetallic effects by increasing the flexural stiffness of the cantilever by increasing its thickness. Owing to its well developed fabrication technologies and excellent structural integrity silicon is the most commonly used material for cantilever biosensors. The sensitivity of silicon cantilever however remains deficient compared to the polymer cantilevers, mainly because of its high stiffness. Therefore, to improve the sensitivity of silicon cantilevers changing the size or shape of the cantilever is another option, and this study investigated both.

The Stoney equation suggests that for a given surface stress the deflection induced in the cantilever is directly proportional to the cantilever length and inversely proportional to its thickness. In other words, by increasing the cantilever length and/or reducing its thickness the deflection can be increased. The advantages of increase in length are two folds. First it helps in inducing higher deflection as suggested by Equation 6 and second, it aids in easy detecting of deflections, because of optical-lever principle. The length of the cantilever however can not be increased arbitrarily because it will also increase the size of the biosensor resulting in loss of device miniaturization. The cantilevers used by Arntz *et al.* [1], a reference model used in this study, have a thickness of 0.5 μm. This thickness can not be reduced further because it is already too thin and further reduction in its thickness would not only affect its structural integrity but also increase its fabrication cost. Therefore, considering the aforementioned constraints pertaining to the cantilever length and thickness change, it is necessary to carry a size change deflection characterization of the cantilever.

**Figure 6.**
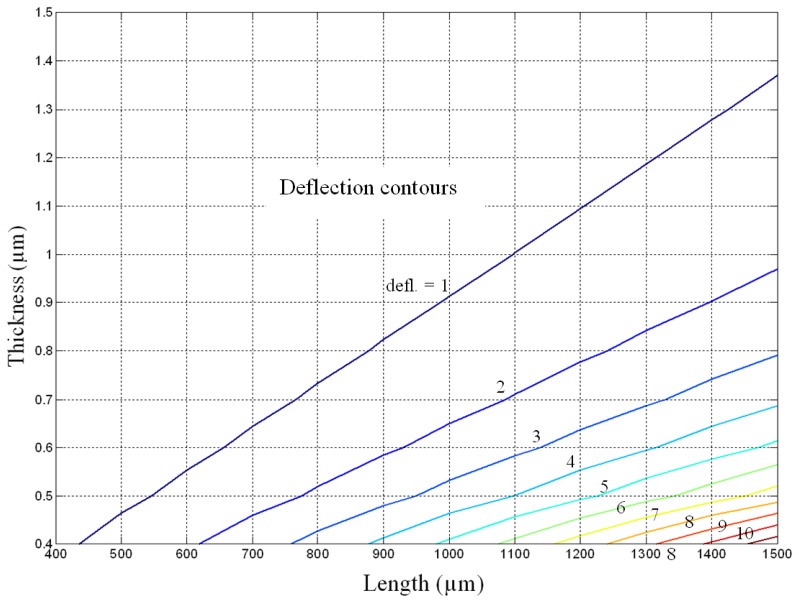
Stoney equation based normalized deflection contour showing relation between the deflection and the length and thickness for a conventional cantilever.

To perform this exercise a deflection contour based on Equation 6 involving the length and thickness of conventional cantilever is plotted (Figure 6). A multipurpose numerical computing and programming language MATLAB was used to plot it. The width of the cantilever is not considered in plotting the contour plot because the width term is intrinsic in defining the surface stress; moreover the surface stress is expressed in terms of force per unit width. The figure shows the relation between the deflection and the length and thickness of the cantilever, for a given surface stress and cantilever material.

From the Figure 6 it is evident that for any given thickness the deflection increases with the increase in cantilever length, but for any given length the induced deflection decreases with the increase in cantilever width. This behaviour is understandable because Equation 6 predicts the deflection is directly proportional to the length square and inversely proportional to the thickness square of the cantilever. To elucidate the contour plot, Figure 6 predicts that the deflection for a conventional cantilever of size 1,500×0.8 μm is about three times that of 1,100×1.0 μm size. In practice however the predictions shown in the figure become less and less reliable as the deflection values exceeds one-half the cantilever thickness, because the deflection then becomes a nonlinear and large deflection problem for which the accuracy of Stoney equation is poor. In large deflection cases the actual deflections are lower than the predicted.

The dynamic analysis of microcantilevers used in biosensors is necessary to predict its accurate deflections induced solely by the surface-stress. In practical applications there can by thermal induced, flow-induced excitations that can interfere with and hence produce noise in deflection signals. To prevent noise, a cantilever should have high natural resonance frequency. The fundamental resonance frequency of a rectangular cantilever beam is given as:

(8) $$f_{0}=\frac{1}{2\pi }\sqrt{\frac{E}{\rho }}\times \frac{t}{l^{2}}$$

where *ρ* is the mass density of the cantilever material. This equation states that the resonant frequency of a rectangular beam is directly proportional to its thickness, and inversely proportional to its length. Therefore, the resonant frequency can be increased by either increasing the thickness and/or decreasing the length. A simplified form of above equation is given as:

(9) $$f_{0}=\frac{1}{2\pi }\sqrt{\frac{k}{m}}$$

where *k* is the spring constant of the cantilever and *m* is its mass. The Stoney equation however predicts a different behaviour, i.e. deflection can be increased by increasing the length and/or decreasing the thickness. In this study, the improved deflection shown by the proposed design is possible mainly because of the reduction in the spring constant of the cantilever. By reducing its cross-sectional area towards the fixed end, we reduced its flexural stiffness to bending which manifests a higher deflection. However Equation 9 suggests that any reduction in spring constant will also decrease its resonant frequency. Therefore, we may conclude that though more sensitive than the conventional the resonant frequency of the proposed design is less. By putting the material and geometric properties of the two designs, Equation 9 can be used to show that *f_0, proposed_* = 0.47 *f_0,conventional_*.

As suggested by Equation 8, the resonant frequency depends on both the material and geometric properties of the cantilever. If we are using silicon cantilevers the reduction in resonant frequency is practically not significant because silicon has excellent mechanical and thermal properties. Due to its high elastic modulus, silicon cantilevers will not be much affected by the external sources of excitation. Polymer cantilevers in contrast can be significantly affected by the reduction in resonant frequency owing to their low elastic modulus. Therefore the resonant frequency of polymer cantilevers should be increased, which can be achieved by increasing their thickness. The proposed design may not be suitable for polymer cantilevers. Hence, for increasing the sensitivity of polymer cantilevers, instead of changing their shape changing the size is a better option. Thus, based on the above discussions on the bimetallic effects, the large deflection behaviour and the interpretations of Equation 9, we can safely conclude that increasing the cantilever thickness is a better way to increase the sensitivity of polymer microcantilevers used in biosensing application. The deflection contour shown in Figure 6 can be efficiently used for this purpose.

**Figure 7.**
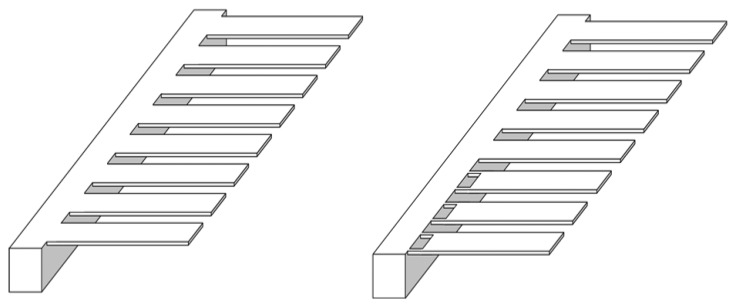
A comparison between old (left) and new (right) microcantilever array biosensors.

For *in vivo* detection we need a sensitive biosensor that can assay analytes in large concentration range simultaneously. For such biomedical applications a new array design is proposed (Figure 7). The figure shows a comparison between conventional and proposed cantilever eight cantilevers array designs. The conventional array design uses eight cantilevers of uniform cross-section. Proposed array design uses a combination of old and news cantilever designs. Since the proposed cantilevers are nearly twice sensitive than conventional, they can be used effectively in assaying target analytes whose solution concentration is comparatively lower. In both the array designs one cantilever type in each can be assigned as a reference for differential deflection readout, which is a popular mean to eliminate noise in deflection signals. The reference cantilever is made passive by depositing buffer materials onto it, and hence it does not participate in the reaction. Thus, we may conclude that by using an array combination of conventional and proposed cantilevers on the same biochip, a high sensitive biosensor can be designed. Such a sensor can simultaneously detect analytes in extremely large, dynamic concentration range.

## 5. Conclusions

Microcantilever array biosensors are becoming increasingly popular in label-free, realtime and simultaneous detection and monitoring of various chemical and biochemical target analytes. The deflections in microcantilever biosensors lie between few tens to few hundreds of a nanometre, which necessitate sophisticated and expensive readout techniques. The ultimate goal of the microcantilever biosensor design and development is to make them sensitive enough to be used in *in vivo* medical applications where accurate, realtime and simultaneous analysis of various clinically important analytes is required. Towards accomplish this goal this study proposed and analyzed a new cantilever design that is about 75% more sensitive than the conventional design. The frequency analysis showed that the natural resonant frequency of proposed design is about half the conventional, i.e. *f_0, proposed_* = 0.47 *f_0, conventional_*. For a given surface stress of 0.05 N/m, the conventional and the proposed cantilever showed maximum deflections of 0.93 μm and 1.62 μm, and induced respectively maximum stresses are 0.41 and 1.12 MPa in the cantilever. This study found that for increasing the sensitivity of microcantilever an increase in the thickness is a better option. Moreover, for increasing the sensitivity of silicon cantilevers shape change is appropriate, whereas for polymer cantilevers size change is more suitable. This work also suggested a deflection contour that can be used in selecting the length and thickness of conventional cantilever for biosensors.

## Figures and Tables

**Table 1.** Comparison between experimental and analysis results.

**Max. Surface Stress (N/m) [1]**	**Max. Deflection (μm)**			
	**Exp.[1]**	**Simulation**	**Stoney (Equation 6)**	**Sader (Equation 7)**
0.05	0.89	0.93	0.83 0.86	
